# Pet Food Palatability Evaluation: A Review of Standard Assay Techniques and Interpretation of Results with a Primary Focus on Limitations

**DOI:** 10.3390/ani5010043

**Published:** 2015-01-16

**Authors:** Gregory C. Aldrich, Kadri Koppel

**Affiliations:** 1 Department of Grain Science and Industry, Kansas State University, Manhattan, KS 66506, USA; E-Mail: aldrich4@ksu.edu; 2 Sensory Analysis Center, Department of Human Nutrition, Kansas State University, 1310 Research Park Drive, Manhattan, KS 66502, USA

**Keywords:** acceptance, palatability, pet food, preference

## Abstract

**Simple Summary:**

Palatability of pet foods is typically measured using a single-bowl or a two-bowl test. While these tests give a general understanding of the liking or preference of one food over another, opportunities exist for further method development.

**Abstract:**

The pet food industry continues to grow steadily as a result of new innovative products. Quality control and product development tests for pet foods are typically conducted through palatability testing with dogs and cats. Palatability is the measure of intake of a food that indicates acceptance or the measure of preference of one food over another. Pet food palatability is most commonly measured using a single-bowl or a two-bowl assay. While these tests answer some questions about the animals’ perception of the food, there are many limitations as well. This review addresses some of these limitations and indicates opportunities for future research.

## 1. Introduction

The pet food industry is a $23 billion enterprise in the USA [1] and $58.6 billion globally in 2011 [2] with an average annual growth of 4%. Innovation and new product development continue to drive the growth of the pet food market. Cat and dog foods account for most of the market share. This growth is supported by continual validation of product performance criteria by animal subjects that consume the foods and also reaffirmation by the pet food purchaser. These performance criteria include pet food sensory properties, such as texture, aroma, and flavor [3]. The pet food industry has relied on several animal models to evaluate pet foods. These models are rapid and relatively inexpensive with the expectation that they provide solid direction and confirmation of the products efficacy.

The models include several key criterions for which pet foods are evaluated on a routine basis such as nutritional adequacy (Association of American Feed Control Officials (AAFCO) feeding protocols), diet digestibility, stool consistency and quality, and palatability [4]. Often times this last item, palatability is all that matters in determining the success or failure of a pet food in the market. There has been a significant amount of product development research applied to this notion of improving pet food palatability. It’s as much a competitive issue as a need for the animal to like the food. For the obvious reason most of this research has been proprietary. As such, a palatability threshold is nearly always a criterion for new product development, it is a common target for product improvements, and it can be quality control measure used to evaluate processing and ingredient options. These all presume that a valid methodology has been established for decision-making. However, many of the techniques used today have provided limited and unreliable information.

There is a clear need to enhance our understanding of the most robust techniques to evaluate palatability. However, unlike sensory analysis for human food products, our pets cannot talk to us to fully describe what it is that they want. Because of this it is difficult for us to discern whether or not they truly like the product. This notion of “liking” is important, because we’re often trying to entice the animal to eat foods that differ substantially from their native foods (e.g., prey). Plus, the owner must be convinced that their pet relishes the meal or they will discontinue purchase. Thus, we’ve resorted to some techniques to monitor the pets’ behavior during a meal to discern whether or not they like a food or have preference for one food over another. Compound this with the industry need for rapid methodologies in order to make decisions and the fact that pets cannot really provide us with direct auditory feedback, and we have a significant challenge to adequately describe this whole notion of palatability. For pet foods the closest corollary to human foods as it relates to sensory analysis would be the human infant and how we must discern whether he “likes” the peas or the liver pate Mom is trying to offer. Parents can typically tell, and so can the so-called pet parents. Unfortunately, this behavior has not been deciphered into quantifiable methods of analysis suitable to academia or industry.

Therefore the objective of this short review is to provide an overview of the techniques currently used to evaluate palatability and to describe where the limitations and potential future research opportunities are. Discussion is provided regarding the validity of these techniques for making product-related decisions and improvement opportunities as it relates to interpretation of the results.

## 2. Animal Factors Effect on Palatability

Feeding behavior of cats and dogs has been summarized in several texts and reviews [5,6,7,8,9,10,11,12,13,14,15,16,17,18] and will be paraphrased below.

While cats (*Felis catus*) and dogs (*Canis familiaris*) live in our homes and have become surrogate children or family members for many owners they differ from humans in many ways. The dog originates as a hunter that courses their prey in organized packs and is capable of eating large quantities of food at the kill in a short time. The dog is also an opportunist for meals and will augment their diet with foraging for anything seemingly edible: From other animals’ feces or scat, to insects, berries, and grass. They are often known to eat carrion and seem to find comfort, if not real enjoyment, in chewing on bones, hides, and other animal parts. Anyone that has a dog that lives part of their day outdoors will attest to some or all of these vestigial behaviors. In the dogs’ mouth are large canines, small premolars, and they lubricate their food with a serous saliva, sans amylase, produced from four primary salivary glands located throughout the oronasal cavity. Not much time is given to masticating and savoring the food during the eating bout: Dogs are known to devour the food in a gluttonous manner and then regurgitate and re-consume at a time and place when away from competing mouths.

The cat is commonly a solitary hunter who lays in wait for their prey to show themselves. Once the kill is made the cat will bolt the food down rapidly and in the case of very small rodents the predator can be seen eating the whole creature intact. For larger prey they will tear flesh and consume in large whole sections. Not much time is given to maceration or savoring the meal. The cat has large canines, small incisors, and premolar carnassial teeth that resemble a serrated knife in appearance. The cat has active olfactory senses. While not as developed as the dogs for hunting and location, the cats olfactory system is tuned specifically to test for novel or untrusted aromas. They will often smell their food exhaustively to ascertain its freshness and safety. The cat is not an ambitious opportunistic eater like the dog, and is selective about what they will finally ingest. In many cases this behavior is learned and imprinted during kitten-hood and will track along family lines. The cat has a similar salivary gland makeup as the dog. The saliva is being produced from four primary glands, and does not produce amylase. The cat has similar taste receptors to the dog, but no functional sweet taste receptors [19,20]. Cats are in essence a prey driven animal commonly referred to as an obligate carnivore for these morphological details, along with many other differences in metabolism (e.g., requirement for preformed Vitamin A, Arachidonic Acid, Taurine, Niacin, *etc.*) [7]. Because of these differences, the cat often seems to be a foreigner in our homes as we offer them a wide array of processed foods and their reluctant consumption behavior supports the perception that they are finicky. However, this behavior may be better understood in light of their food-nutrition needs with context for how the cats “see” their food world. Therein lies the challenge with the palatability test. How to discern the mind of the dog or cat?

## 3. Palatability Assessment in Pets

Taste describes the sensation arising from the stimulation of chemoreceptors (taste buds) located in the oral cavity. Odor or smell comprised of the volatile components in food affect the olfactory receptors (olfaction) located in the nasal cavity. Flavor connotes the combined perception of the taste and olfactory receptors in the oral, nasal and laryngeal cavities. Palatability is the perception derived at the time food is consumed and accounts for the flavor and the animals’ perception of the appearance, temperature, size, texture, and consistency and perhaps prior experiences [5,10]. The National Research Council’s guidelines [14] adapted from [21] give the working definition of palatability as the “physical and chemical properties of the diet which are associated with promoting or suppressing feeding behavior during the pre-absorptive or immediate post absorptive period”. In other words the unconditioned response before any metabolic or condition effects on food intake could occur. This simplifies the use of palatability relative to the more commonly identified notion that it is the sum of taste that is pleasing, a hedonic or pleasurable response that takes into account the pre- and post-absorptive behavior, aversions, and satiety. The more restricted understanding of palatability that isolates intake and appetites for certain nutrients from a metabolic perspective is important. However, it fails to capture the whole picture associated with food. By restricting palatability to food as mere nutrient delivery device it discounts the more vital elements of palatability that involve the human-animal interaction, the animal’s previous experiences. An expanded definition of palatability as a more holistic perspective would include the food in total, animal factors, time and prior exposure, and human and environmental influences.

There are two classes of palatability tests: Consumption and non-consumption tests [22]. The non-consumption tests are autonomic or conditioned response tests such as a Pavlovian response of the dog to a meal, and the other are instrumental or operant conditioning tests like a Skinner box test where the animal learns to associate an action with a reward (or denial of the reward). A more recent description has been the cognitive palatability assessment protocol by Araujo and Milgram [23] and Araujo *et al.* [24] that relies on discrimination learning by dogs for 3 objects (or foods) at a time (see Section 3.3).

The more common industrial techniques are “consumption” tests that measure intake: One a monadic or single-bowl “acceptance” test and the other a two-pan or forced choice “preference” test [12,21]. The goal in each is to understand the acceptability or preference of a food. Acceptability could be defined as the consumption of an adequate amount of calories in a food item to sustain weight and performance without regard to any taste, olfactory consideration. Whereas, preference is the incremental or graded choice for one item over another and infers that something is being compared. Aroma is the sum of the olfactory cues from the food and environment and in the dog and cat is accentuated by the vomeronasal organ (Jacobson’s organ), which is an anatomical adaptation to the olfactory sensing organ that amplifies aroma. It may be influenced by moisture and temperature. The behavior we often track to describe a response to the foods aroma is the “first-choice” response. This is the food first approached and consumed by the animal under the conditions of a given protocol. At times, certain animals will refuse an item of food that others in their contemporary group clearly are pleased with—This may be a “conditioned aversion” or a learned refusal behavior derived from earlier exposure. We often use terms such as neophobia—Fear of new or novel, or neophilia—Love of new or novel, to describe some of the responses to new foods offered to the animal. These are more common descriptions of food offered to the cat than dogs; but ultimately apply to both.

### 3.1. The Single-Bowl Test

In the monadic or single-bowl test a food is weighed and offered to the animal [22]. Food intake is determined by difference from initial food on offer and orts (or leftovers) after a specified period of time. This can be monitored under normal feeding parameters singly or for multiple feedings per day and is repeated for several days, typically 5 days or longer. Often the food will be switched to a new item and the steps repeated. In this case intake can be compared between the periods in a switchback design. For experimental purposes multiple dogs or cats can be used and balanced by period in order to eliminate any environmental influences. The results indicate whether the animal, when presented with no choice, refuses a new food or with multiple periods consumes more or less of one food relative to the other. The benefit to this test is that it mimics in many ways the home setting where the animal does not have a choice, *i.e.*, is served one food for a meal. It is effective at identifying products that are completely unacceptable due to an off-flavor, aroma, or texture, and (or) might be prone to cause some digestive feedback over an extended period of time. This test provides a measure or inference regarding “acceptance” but does not yield information about preference, degree of liking, or any other hedonic aspect of the food. Furthermore, cats and dogs adapt to new or different foods in dissimilar manners and this technique does not take into account these specie-specific differences. The cost of the test can be relatively low and the use of 8–10 animals is typically sufficient to detect a trend. It may be more suitable for a “worst-case scenario” evaluation of a product, or even as a quality check for customer service purposes should there be complaints about a certain food. However, the method does not supply adequate information for a company to use for marketing claims, for flavor direction, or product improvement activities.

This test is typically conducted in a kennel setting, but could also be used in an in-home-use-test (IHUT) situation. Any breed and size animal can be used. Taste discrimination need not be a qualification for an animal’s participation. However, care should be taken that food amounts offered do not exceed normal daily caloric intake (plus 10%) or animals can become overweight, which may influence interpretation of the results. Further, research has shown that kennel animals do not necessarily react to foods in the same way as home animals [25]. Results from animals evaluated in a home setting may vary more in their acceptance toward foods because of differences in prior feeding. This may be overcome with the use of a control diet for a certain period of time (4–5 days) prior to feeding the test product.

This monadic test simply measures daily intake of the test food. While this is a good indicator of acceptance, it seems there may be considerable opportunity to develop this method further and include other indicators of liking to this test. These indicators may include heart rate, pupil dilation, respiration rate, activity level, body movements, eating rate, or other parameters. Some research has been done on facial indicators of food acceptance and rejection by cats [26]. Technology that helps monitor some of these indicators, such as activity level and heart rate, has been developed [27]. In addition, recording and analyzing videos of the feeding situation may provide additional insight to the relative liking of the test food.

#### 3.1.1. Single-Bowl Test: A Case Study

**Figure 1:**
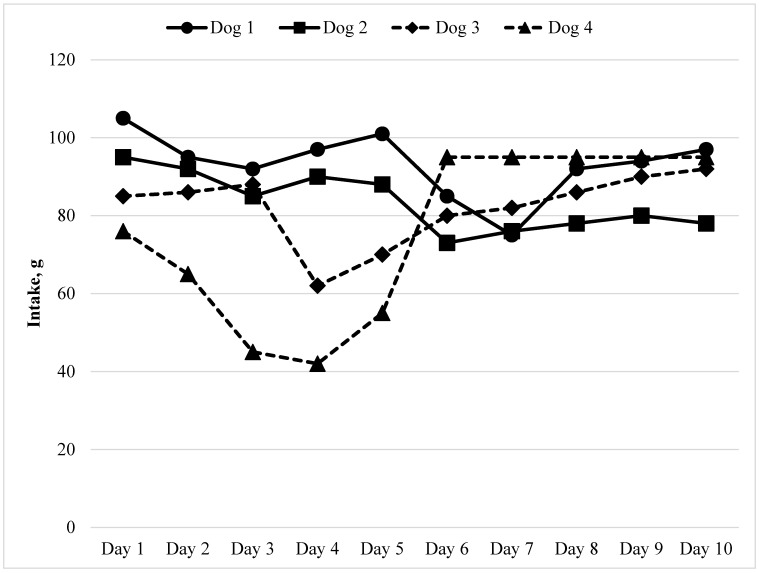
Food intake (g) in a single-bowl test.

An example of a single bowl test is shown in Figure 1. Using hypothetical data 4 small dogs are each presented with one bowl of food that should support their daily caloric needs. Dogs 1 and 2 are presented with Food 1 on days 1–5 and Dogs 3 and 4 are presented with Food 2 on days 1–5. Then Foods 1 and 2 are switched for the dog pairs on days 6–10. From this evidence it can be seen that Food 2 is novel and there is some initial adjustment to its consumption by each of the dogs. The dogs accepted Food 1, but overall consumption was 81% of that of the Food 2. An intake ratio [Food 1/(Food 1 + Food 2)] of 0.50 would represent equivalent consumption. In this case the Intake ratio (IR) for Food 1 was greater than 0.50 at 0.55 which would lead one to conclude that it was consumed at a greater quantity after accounting for the period effect (Table 1).

**Table 1:** Summarized food intake by dog and Intake Ratio (IR).

Dog	Food 1, g	Food 2, g	IR *
1	490	443	0.53
2	450	385	0.54
3	430	391	0.52
4	475	283	0.63
Total intake, g	1845	1502	0.55
Average daily intake/dog	92.25	75.1	

* IR = Food 1/(Food 1 + Food 2).

### 3.2. The Two-Bowl Test

The most common method for palatability evaluation is a split-plate or two-bowl forced choice evaluation [21,28,29]. This may seem most logical in the development of new products when attempting to create an improvement of one product over another; wherein the intent is to determine whether the animal has a preference.

In this methodology two foods are placed in their respective bowls and presented simultaneously to the animal. Thus, unlike the monadic test there is a choice. The animal is allowed to smell the food and then the bowls are placed simultaneously before the animal for consumption. The technician will monitor which of the two foods have been approached first, and from which the first bite was taken. This “first bite” is commonly assumed to be related to the aromatic characteristics of the food. The bowls are left with the animal for a set duration of time, commonly 15 to 30 min or until one of the bowls has been completely consumed. The amount of food offered in each bowl should be sufficient for the animals’ daily caloric intake. The test is administered in the morning after an overnight fast. The total consumption out of the two respective bowls is quantified to determine whether one bowl or the other was consumed in a greater proportion. Thus, the possible results are preference for Food A, Food B, neither, or a portion from both. It is preferable that the animals have sniffed and even selected from both bowls to demonstrate that each was tested.

Animals used for palatability testing should be qualified before feeding studies. They should be healthy with no predisposing illnesses or behavioral problems that preclude them from providing objective and balanced results. Case in point, animals in heat and intact males with aggressive tendencies may need to be excluded. Animals with timid or aggressive tendencies may need to be separated and not housed in cages side-by-side. The cats and dogs should be validated prior to conduct of product or process development work. This can take on several phases starting with adaptation to the method, followed by an obvious choice test, and finally a null test. The adaptation to the method is simply a way for the animals to become acclimated to the environment and routine of the test, to the way the food will be presented, and with the presence of a technician nearby during the meal presentation. The obvious choice test is used to validate that the animal has the ability to discriminate when there is a known and intentional difference in the foods. This can be done by using a complete diet *vs.* one that lacks fat (for dogs) or lacks flavor coating (for cats or dogs) and determining that the animal chose most of their portion of food from the more flavored bowl. Once this test has been passed one can evaluate the animals in a null test in which the same food is offered in each bowl. This can lead to confusing results, so interpretation should be limited to understanding if the animal has a side-bias (left or right bowl preference) and understanding if this is a strong bias, minimal, or has foundation in some other environmental factor (as noted above). Any dog or cat, small or large, popular or rare breed, can be used for palatability. Even the more robust eaters in the dog world like the Labrador Retrievers, if they have the ability to discriminate, can be effective in a two-bowl palatability test. While the gluttonous eater may be able to discriminate, they can be harder to manage in rooms with a large number of animals and few technicians. The variation among cat breeds is not as huge as among dog breeds, although significant difference exists in sizes and other physiological characteristics, like muzzle shape. There’s a vast difference among dog breeds, though, from the “toy” breeds to “working” breeds. No evidence exists to suggest that any breed has more ability to discriminate one food over another, but it stands to reason that there might be breed differences. The goal for any worthy conclusions from a given project is that the sub-population selected for the study is representative of the population as a whole, and that the inferences can be applied uniformly across all cats or dogs when the food is introduced into the broader market and into a home. We know from experience and from literature [25] that how the food performs in the home can be quite different from the kennel results. In short, because the kennel results look solely at the animals’ intake data; whereas, in the home other behavior (visual, postural, and activity) queues are factored into the pet-owners conclusions.

The statistical analysis for first approach and first choice can be evaluated by chi-square analysis. For total consumption and consumption ratio the means can be separated by use of a t-test. The amount of food consumed can be evaluated as total grams consumed of each bowl and comparing those to each other. This works satisfactorily if the animals in the test are of roughly the same weight. Alternatively the data can be compared as an intake ratio where each of the foods consumed is expressed as a proportion of the total food consumed; typically that number would be expressed as a decimal. This is more appropriate when the animals differ in size and weight because it factors for the proportion consumed rather than the absolute amount. Another estimate the “preference ratio” can be determined with a few assumptions. First for those animals consuming roughly equal amounts of both foods, they are in essence not making a choice and are factored out. Those animals selecting at least ⅓ or better for one food or the other are said to have a preference (an IR less than 0.33 or greater than 0.67). They are “counted” for one food or the other and expressed as a ratio of A: No Choice: B. This provides a deeper inference regarding choice when two foods are of very similar flavor profiles and intensities.

This series of analysis can be conducted for 2 days (or two day multiples) with the bowls switched between days to override any influence of handedness or side bias. Some research groups will provide one day as an adaptation phase before collecting data on days 2 and 3 to account for this side bias challenge, and as an adaptation period to a new food. Other groups will allow for longer feeding times anywhere from 4 to 6 days with the idea that increasing the number of days will improve the reliability of the test with more observations. The additional days however are not additional observations, but rather additional measures of the same animal: A repeated measure.

This type of measurement allows one to evaluate the palatability or preference of one food over another when there is truly an obvious choice; especially, if side bias between or among the animals has been taken into account. It does not consider animals that cannot make a choice or discriminate, or those that find the food to be equivalent. This method also fails to tell us anything about whether or not the pet actually likes the food, if both foods are undesirable, or if both foods were equally well liked. Food on offer and contact time allowed can be a challenge to manage in these tests as the goal is to provide the food in an amount in each bowl that would provide sufficient calories for the day (or feeding period). However, by doing so the animal is offered twice the amount of food they would need for a day. If the animal chooses to eat a significant amount of each they could begin to gain weight.

The number of animals included in the test is also an important consideration. In previous years it was common in a split plate test to use 10 animals and conduct the test for 5 to 6 days. That way the experimenter would collect 50 to 60 observations. However, with the resolution that this is actually providing repeated observations per animal for the same measure; it became less efficient to conduct the test in this manner. So, most research groups have moved to feeding 20 animals for 2 or 4 days. This provides more true observations of the experimental unit (the animal) and provides a quick view into whether the animal has a preference for the particular food in question. This is great for competitive analysis or new product development. However, it suffers when the foods are similar such as production tests or quality control checks. It can be effective when evaluating new flavor systems and intentional product enhancements. However, it does not tell in the short-term tests whether there might be flavor fatigue, or if the food has some longer-term nutritional feedback on taste and satiety. Further, the two-bowl test fails to indicate exactly what the animal might be liking or not liking about a particular food. It is solely dependent on the reference standard the food that is placed in the “other” bowl. However, it does not elucidate any particular preference for a specific element in a complex food. Systematic replacements or repletion studies can help to study these issues; but that adds significantly to the timeline as well as testing costs.

#### 3.2.1. Two-Bowl Test: A Case Study

An example of a two-bowl test is shown in Table 2. The standard assay of feeding diets A and B over 2 consecutive days was used. In this example, Food A was chosen first more often than Food B (6:4), and was reversed the second day (4:6). One would conclude that there was no difference between the two foods for this factor and infer that the aroma of the two foods did not differ substantially. Regarding consumption, two of the dogs appear to be side-biased with a majority or all of their consumption from the right bowl (Dog 3 and 6) or left bowl (Dog 9). This has the effect of biasing the data to the middle (0.5 intake ratio). For these hypothetical results Food A was consumed in slightly lesser amount than B (1967 *vs.* 2050 g; or 0.9595:1). There is a wide range in dog sizes as demonstrated by the vast differences in food intake, so that consumption ratio is the preferred method to report the results (for Food A was 0.4897 or less than 0.5 indicating that the panel chose more of B). The t statistic (t = 0.1366) is less than the t_0.05_ = 2.262 [for 9 degrees of difference in a two-tailed t-test] therefore, the values are similar. In this case, whether we review the data for total consumption or IR, the conclusions are the same; the foods are similar. If one goes one step further to evaluate the preference ratio the preference ratio count would be 2:8:0; wherein, 2 dogs preferred food A with an IR greater than 0.67, 8 dogs had an IR between 0.33 and 0.67, and 0 dogs had an IR of 0.33 or less.

**Table 2:** Food intake (g) in a two-bowl test.

	Day 1	Day 1	Day 2	Day 2	Total A	Total B	A/(A + B)
Dogs	A	B	B	A			
1	75 *	25	60 *	40	115	85	575
2	50	20 *	15	72 *	122	35	777
3	10	300 *	25	415 *	425	325	567
4	65 *	30	50 *	10	75	80	484
5	55 *	5	75 *	10	65	80	448
6	0	600 *	0	700 *	700	600	538
7	150	150 *	65 *	65	215	215	500
8	60 *	15	55 *	15	75	70	517
9	40 *	5	35 *	0	40	40	500
10	95 *	10	10	40 *	135	20	871
Subtotal	600	1160	890	1367	1967	2050	4897
FC **	6	4	6	4			

* First choice of food. ** FC—Sum of first choices made by an animal.

### 3.3. Other Methods for Determining Palatability

A variation of the two-bowl method that has been presented in the literature includes the operant lever-press test [30]. In this test the animals would be trained to turn a lever with their nose based on what food they wanted more of. Rashotte *et al.* concluded that this test does not always provide similar results as the two-bowl test. In addition this method requires additional apparatus and testing systems setup [30]. Another version of this test requires the animals to press down a lever in order to acquire more food [31]. While these authors also point out the lengthy training that is required to conduct this method, they also indicate that the method provides similar results to the two-bowl test.

According to Araujo *et al.* [24] a cognitive palatability assessment protocol may provide similar results with less variation and fewer test animals than the two-bowl test. In this technique the test animals are provided simultaneously 3 choices: Test food A, test food B, and a control. From these choices the animal is supposed to select 1. This method does not simulate real-life conditions. In addition this method is limited to foods with an obvious difference and to dogs exclusively with a higher functioning cognitive ability. It does require fewer animals and may isolate preference from post-ingestive feedback and satiety effects. This work may provide insights for future development of more meaningful techniques to assess palatability, but for now most of these operant tests are not the most common techniques to quantify food preferences of dogs or cats. However, it would be interesting to test this method in a modified format in a real-life situation using home animals that do not have any training. Home animals could probably successfully compare 3 or 4 test products side-by-side and establish a first choice, and a second choice, based on aromatic evaluation. Additional input could be gathered by monitoring the animal’s body language and other metrics.

### 3.4. Opportunities for Further Research

To assess palatability the single-bowl and the two-bowl methods are most common. The question though is whether these techniques really provide a good assessment of “liking” or “preference” by the pet and “acceptance” by the pet owner. The work of Smith *et al.* [29] demonstrated that measuring consumption continuously in one and two bowl tests could demonstrate adaptation and vigor of meal consumption, but did not reveal any more about the individual animal preferences. Some studies have proposed alternative techniques to be used instead of single-bowl or two-bowl methods to determine palatability. These more conditioned response type studies using operant testing can provide some limited value to our understanding of preference and liking. However, they can be very time consuming, rely on very specialized facilities, and trained animals and technicians. In the end, these tests can be directional, but remove the influence of the animal’s surroundings and the human-owner factor.

Factoring in the pet owner the use of human sensory analysis technique described by Koppel [32]; Koppel *et al.* [33]; Di Donfrancesco *et al.* [34], and Koppel *et al.* [35] can provide some insight into the taste, aroma, and textural context of pet foods. However, these techniques have yet to be validated against animal models. Connecting these factors would provide significant insight and understanding of what the animal models are telling us.

Some additional insight might be gained by exploring variability among home pets, as they are the ones consuming the commercial products. Variability in preferences may be affected by previous exposure to different foods, feeding situation, family structure, and individual factors such as gender, age, breed, and health condition. While there is a desire to use a method that provides reliable, repeatable, accurate results from one product and animal group to the next, this level of veracity is generally considered outside the prevue of the method for a single test. We know that kennel dogs do not necessarily track with home based dogs [16], that early neonatal imprinting occurs [36] and there is novelty as well as fatigue effects [8]. Because of these and many other factors inherent with interpreting animal sensory or intake responses to a food item, researchers will often run concurrent tests, at multiple research facilities, and over a wide time frame in order to confirm their results. Questions such as if there is an impact of multiple-choice tests (with more than two options) efficacy with animals who have more “experience” with different foods than with animals who have mostly been fed one single food throughout their lives? Would an animal, which is fed in a highly competitive environment, make different choices when being fed alone? Does the health condition affect palatability of food and many others remain.

In order to answer these questions a different approach to researching this topic should be developed. An approach, which includes both intrinsic (such as gender, breed, age, weight, personality, *etc.*) and extrinsic (such as household type, family type, activity level, country, climate, *etc.*) animal factors, should be included in the model. In addition the pet food development process needs to consider multiple contributing factors, such as the influence from ingredients, formulation, processing, coating, and packaging to name a few [37]. As we gain a better perspective on the complex issue of measuring palatability for our pets’ better controls over food allowances, obesity, and other food related matters would be improved.

## 4. Conclusions

Palatability for dogs and cats is the measure of intake of a food that indicates acceptance or the measure of preference of one food over another. Pet food palatability is most commonly measured using a single-bowl or a two-bowl assay. While these tests answer some questions about the animals’ perception of the food, there are many limitations, but also opportunities for future research in developing methods that would help understand palatability-related issues or provide better models to predict cat and dog food selection.

## References

[1] APPA 2014. Pet Industry Market Size & Ownership Statistics: Estimated 2014 Sales within the U.S. Market. http://www.americanpetproducts.org/press_industrytrends.asp.

[2] Transparency Market Research 2014. Global Pet Food Market to be Worth US $74.8 Billion by 2017. http://www.transparencymarketresearch.com/pressrelease/pet-food-market.htm.

[3] Taylor J. (2014). Good taste: Petfood palatability update 2014. Petfood Ind..

[4] AAFCO Association of American Feed Control Officials The Business of Pet Food. http://petfood.aafco.org/caloriecontent.aspx.

[5] Kitchell R.L. (1978). Taste perception and discrimination by the dog. Adv. Vet. Sci. Comp. Med..

[6] Bradshaw J.W.S. (1991). Sensory and experiential factors in the design of foods for domestic dogs and cats. Proc. Nutr. Soc..

[7] Bradshaw J.W.S., Goodwin D., Legrand-Defretin V., Nott H.M.R. (1996). Food selection by the domestic cat, an obligate carnivore. Comp. Biochem. Physiol..

[8] Stasiak M. (2002). The development of food preferences in cats: The new direction. Nutr. Neurosci..

[9] Kvamme J.L., Kvamme J.L., Phillips T.D. (2003). Section IV, Chapter 1: What is palatability?. Petfood Technology.

[10] Bradshaw J.W.S. (2006). The evolutionary basis for the feeding behavior of domestic dogs (*Canis familiaris*) and cats (*Felis catus*). J. Nutr..

[11] Houpt K.A., Smith S.L. (1981). Taste preferences and their relation to obesity in dogs and cats. Can. Vet. J..

[12] Thombre A.G. (2004). Oral delivery of medications to companion animals: Palatability considerations. Adv. Drug Deliver. Rev..

[13] Zaghini G., Biagi G. (2005). Nutritional peculiarities and diet palatability in the cat. Vet. Res. Commun..

[14] (2006). NRC 2006. Nutrient Requirements of Dogs and Cats. Animal Nutrition Series. National Research Council of the National Academies.

[15] Hullar I., Fekete S., Andrasofszky E., Szocs Z., Berkenyi T. (2001). Factors influencing the food preference of cats. J. Anim. Physiol. Anim. Nutr..

[16] Griffin R.W., Beidler L.M. (1984). Studies in canine olfaction, taste, and feeding: A summing up and some comments on the academic-industrial relationship. Neurosci. Biobehav. Rev..

[17] Case D.P., Case L.P., Carey D.P., Hirakawa D.A., Daristotle L. (2003). Chapter 18: Evaluation of Pet Foods. Canine and Feline Nutrition: A Resource for Companion Animal Professionals.

[18] Crane S.W., Moser E.A., Cowell C.S., Millica J., Stout N.P., Romano P., Crane S.E., Hand M.S., Thatcher C.D., Remillard R.L., Roudebush P., Novotny B.J. (2010). Chapter 2: Commercial Pet Foods. Small Animal Clinical Nutrition.

[19] Li X., Li W., Wang H., Bayley D.L., Cao J., Reed D.R., Bachmanov A.A., Huang L., Legrand-Defretin V., Beauchamp G.K. (2006). Cats lack a sweet taste receptor. J. Nutr..

[20] Jiang P., Josue J., Li X., Glaser D., Li W., Brand J.G., Margolskee R.F., Reed D.R., Beauchamp G.K. (2012). Major taste loss in carnivorous mammals. Proc. Natl. Acad. Sci. USA.

[21] McArthur L.H., Kelly W.F., Gietzen D.W., Rogers Q.R. (1993). The role of palatability in food intake response of rats fed high-protein diets. Appetite.

[22] Griffin R.W., Kvamme J.L., Phillips T.D. (2003). Section IV: Palatability. Petfood Technology.

[23] Araujo J.A., Milgram N.W. (2004). A novel cognitive palatability assessment protocol for dogs. J. Anim. Sci..

[24] Araujo J.A., Studzinski C.M., Larson B.T., Milgram N.W. (2004). Comparison of the cognitive palatability assessment protocol and the two-pan test for use in assessing palatability of two similar foods in dogs. Am. J. Vet. Res..

[25] Griffin R.W., Scott G.C., Cante C.J. (1984). Food preferences of dogs housed in testing-kennels and in consumers’ homes: Some comparisons. Neurosci. Biobehav. Rev..

[26] Van den Bos R., Meijer M.K., Spruijt B.M. (2000). Taste reactivity patterns in domestic cats (*Felis silvestris catus*). Appl. Anim. Behav. Sci..

[27] Best Dog Activity Monitor: Fitbark *vs.* Tagg *vs.* Whistle. http://www.caninejournal.com/dog-activity-monitor/.

[28] Hutton J. (2002). Palatability: Two-bowl to twin feeder. Feed Manag..

[29] Smith J.C., Rashotte M.E., Austin T., Griffin R.W. (1984). Fine-grained measures of dogs’ eating behavior in single-pan and two-pan tests. Neurosci. Biobehav. Rev..

[30] Rashotte M.E., Foster D.F., Austin T. (1984). Two-pan and operant lever-press tests of dogs’ preference for various foods. Neurosci. Biobehav. Rev..

[31] Houpt K.A., Hintz H. (1978). Palatability and canine food preferences. Canine Pract..

[32] Koppel K. (2014). Pet food sensory analysis. J. Sci. Food Agric..

[33] Koppel K., Gibson M., Alavi S., Aldrich G. (2014). The effects of cooking process and meat inclusion on pet food flavor and texture characteristics. Animals.

[34] Di Donfrancesco B., Koppel K., Chambers E. (2012). An initial lexicon for sensory properties of dry dog food. J. Sens. Stud..

[35] Koppel K., Adhikari K., di Donfrancesco B. (2013). Volatile compounds in dry dog foods and their influence on sensory aromatic profile. Molecules.

[36] Hamper B.A., Rohrbach B., Kirk C.A., Lusby A., Bartges J. (2012). Effects of early feeding experience on food acceptance in a colony of adult research cats: A preliminary study. J. Vet. Behav..

[37] De Groot J., Schrijver I. (2008). Pet food palatability needs multi-factorial approach. Feed Tech..

